# Supplementary material for: Molecular diagnosis of intestinal protozoa in young adults and their pets in Colombia, South America
Source: PLoS One. 2023 May 23;18(5):e0283824. doi: 10.1371/journal.pone.0283824 (PMC10204978; doi:10.1371/journal.pone.0283824)
Supplement: S1 Appendix — (PDF) [file pone.0283824.s001.pdf]

**S1 Appendix. Gel images**

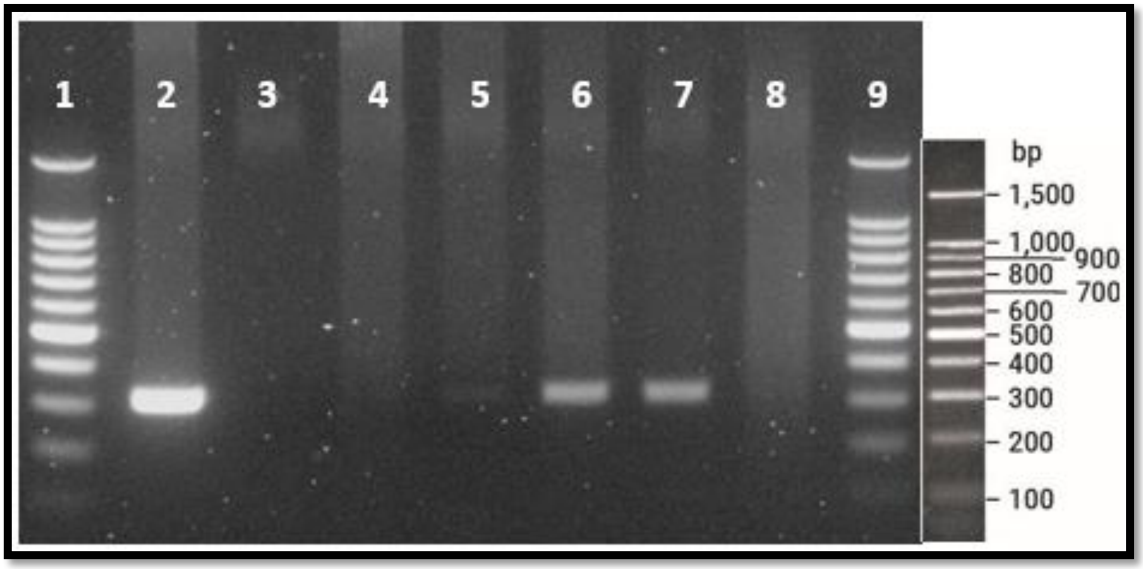


S1.1 Gel electrophoresis of monoplex PCR for *Blastocystis* spp. Lanes 1 and 9: 100 bp DNA marker (Promega). Lane 2 positive control with amplicon size 310 bp. Lane 3: negative control. Lanes: 4-8 patient samples. Samples 5 -7 were positive and samples 4 and 8 were negative.

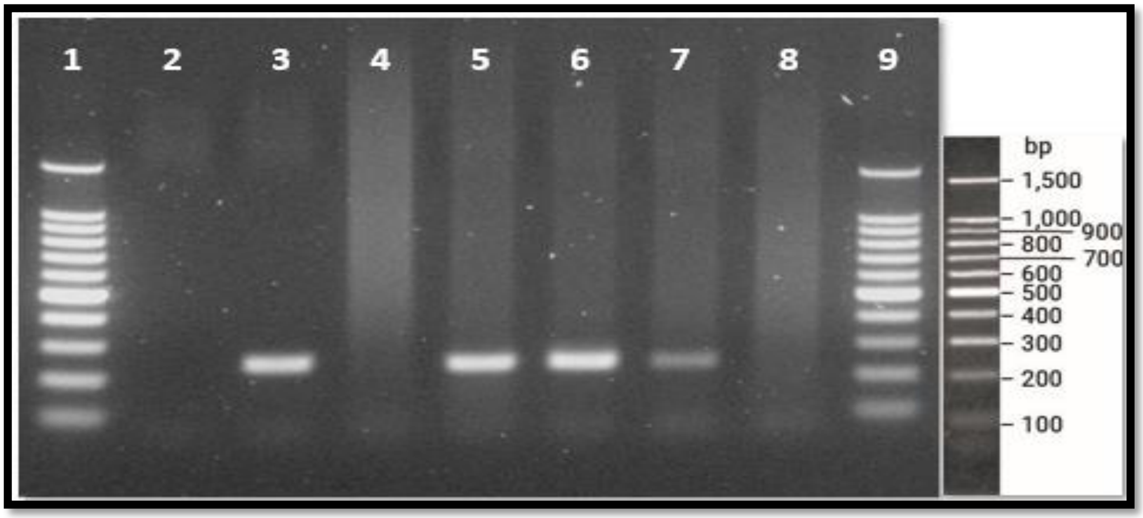


S1.2 Gel electrophoresis of monoplex PCR for *Cryptosporidium* spp. Lanes 1 and 9: 100 bp DNA marker. Lane 2: negative control. Lane 3 positive control with amplicon size 240 bp. Lanes 4 – 8: patient samples. Samples 5 -7 were positive and samples 4 and 8 were negative.

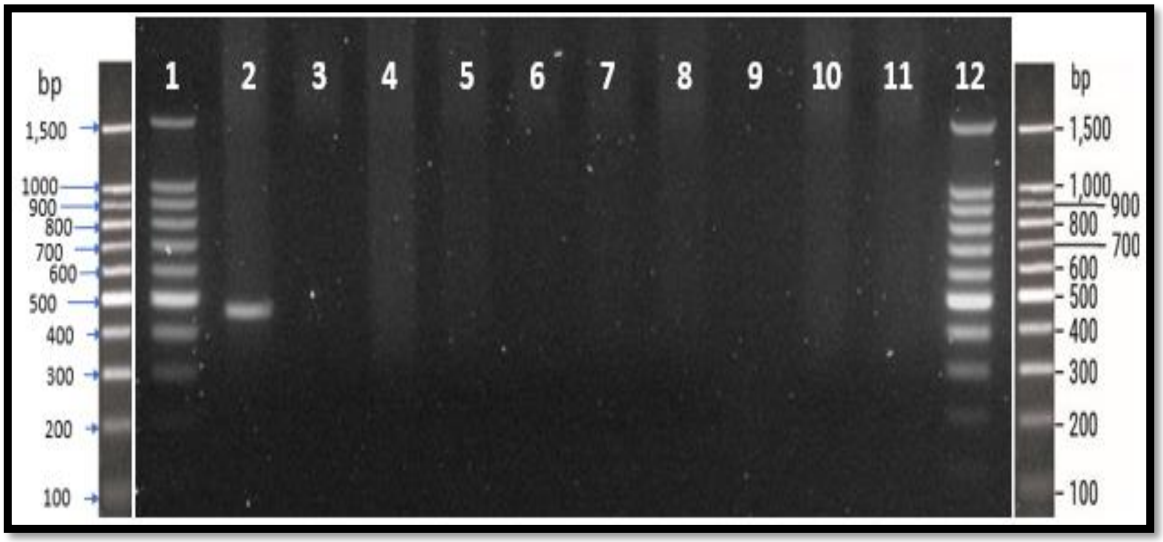


S1.3 Gel electrophoresis of monoplex PCR for *Giardia intestinalis*. Lanes 1 and 12: 100 bp DNA marker. Lane 2 positive control showing a 463 bp amplicon. Lane 3: negative control. Lanes 4 – 11: negative samples.

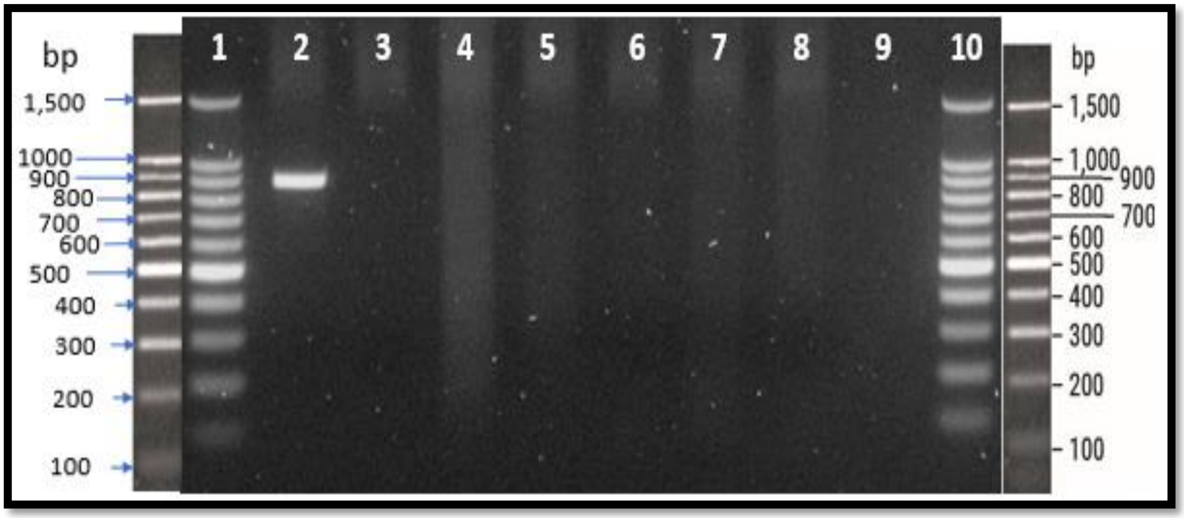


S1.4 Gel electrophoresis of monoplex PCR for *Dientamoeba fragilis*. Lanes 1 and 10: 100 bp DNA marker. Lane 2: positive control (synthetic DNA) showing a 850 bp amplicon. Lane 3: negative control. Lanes 4 – 9 negative samples

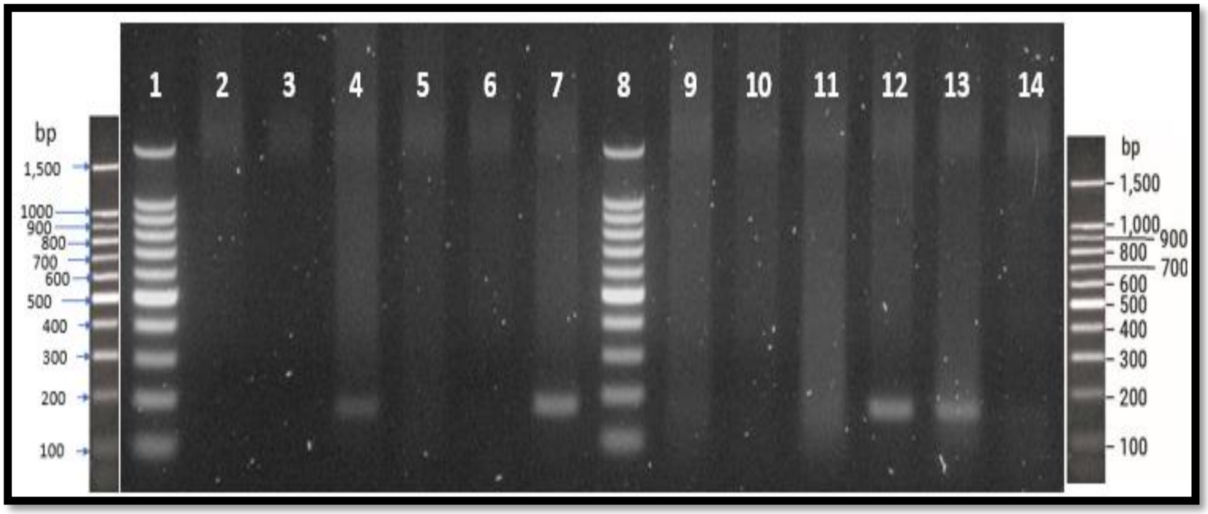


S1.5 Gel electrophoresis of monoplex PCR for *Entamoeba dispar*. Lanes 1 and 8: 100 bp DNA marker. Samples: lanes 3-7 and 9-14. Lane 3: negative control. Positive samples: lanes 4, 7, 12 and 13 show a 174 bp amplicon. Negative samples: 3, 5, 6, 9-11 and 14.
