# Supplementary material for: Molecular diagnosis of intestinal protozoa in young adults and their pets in Colombia, South America
Source: PLoS One. 2023 May 23;18(5):e0283824. doi: 10.1371/journal.pone.0283824 (PMC10204978; doi:10.1371/journal.pone.0283824)
Supplement: S2 Appendix — (PDF) [file pone.0283824.s002.pdf]

S2. GenBank accession numbers

Sequence /sample number	Genebank accession number
SUB11546173 PI006	ON668107
SUB11546173 PI016	ON668108
SUB11546173 PI019	ON668109
SUB11546173 PI038	ON668110
SUB11546173 PI049	ON668111
SUB11546173 PI063	ON668112
SUB11546173 PI092	ON668113
SUB11546173 PI110	ON668114
SUB11546173 DisPI097	ON668115
