# Supplementary material for: Molecular diagnosis of intestinal protozoa in young adults and their pets in Colombia, South America
Source: PLoS One. 2023 May 23;18(5):e0283824. doi: 10.1371/journal.pone.0283824 (PMC10204978; doi:10.1371/journal.pone.0283824)
Supplement: S1 Raw images — (PDF) [file pone.0283824.s003.pdf]

S1_raw_ images

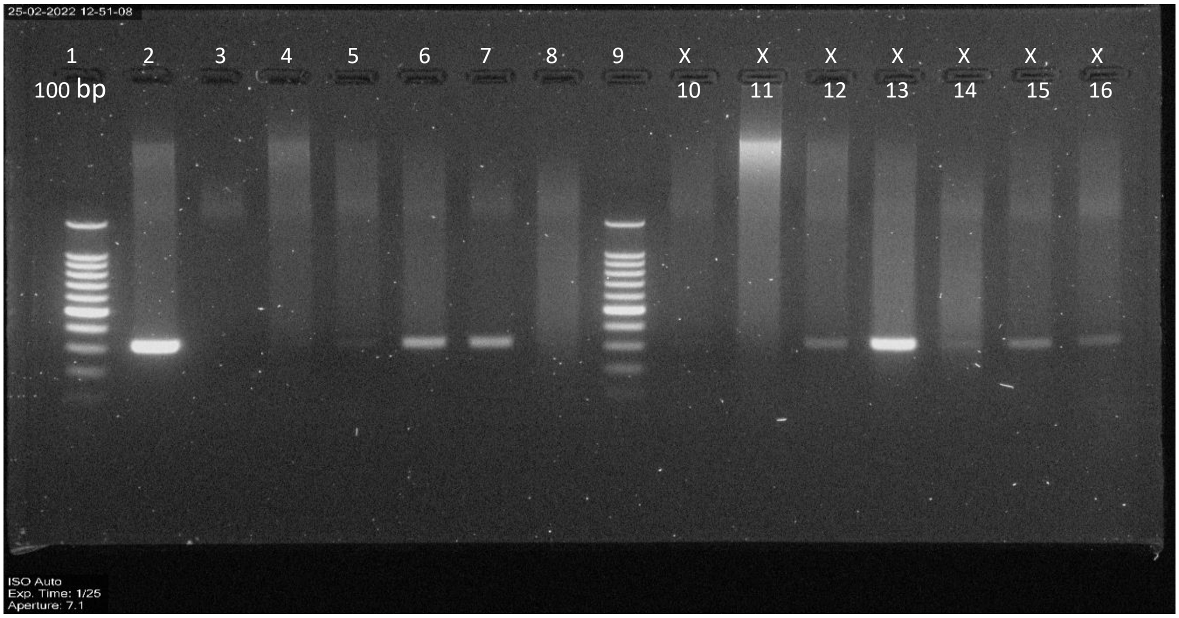

S1.1 Gel electrophoresis of monoplex PCR for *Blastocystis* spp. Lane 1 and 9: 100 bp DNA marker (Promega). Lane 2 positive control with amplicon size 310 bp. Lane 3: negative control. Lanes: 4-8 and 10-16 are patient samples. Samples 5-7, 10 and 12- 16 were positive and 4, 8, 11 were negative. Capture method: Gel Doc system LED FastGene FAS-DIGI PRO (NIPPON Genetics Europe). Lanes 1-9 were included in Appendix 1, Fig S1.1.

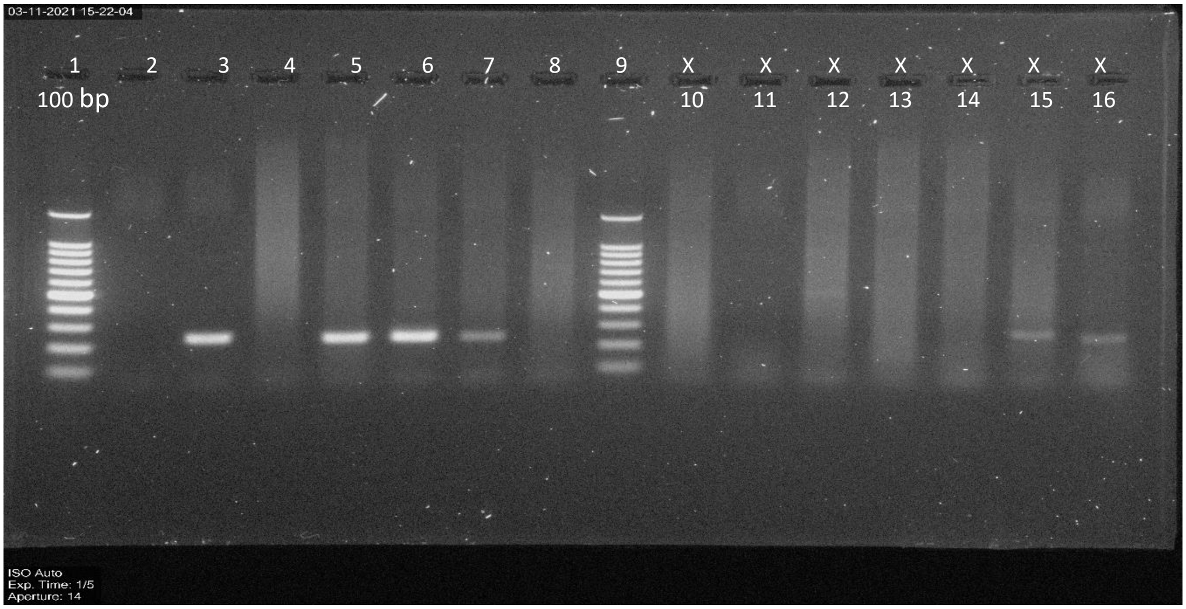

S1.2 Gel electrophoresis of monoplex PCR for *Cryptosporidium* spp. Lane 1 and 9: 100 bp DNA marker. Lane 2: negative control. Lane 3 positive control with amplicon size 240 bp. Lanes 4 – 8 and 10-16 are patient samples. Samples 5 -7, 15, 16 were positive and 4, 8, 10-14 were negative. Capture method: Gel Doc system LED FastGene FAS-DIGI PRO (NIPPON Genetics Europe). Lanes 1-9 were included in Appendix 1, Fig S1.2.

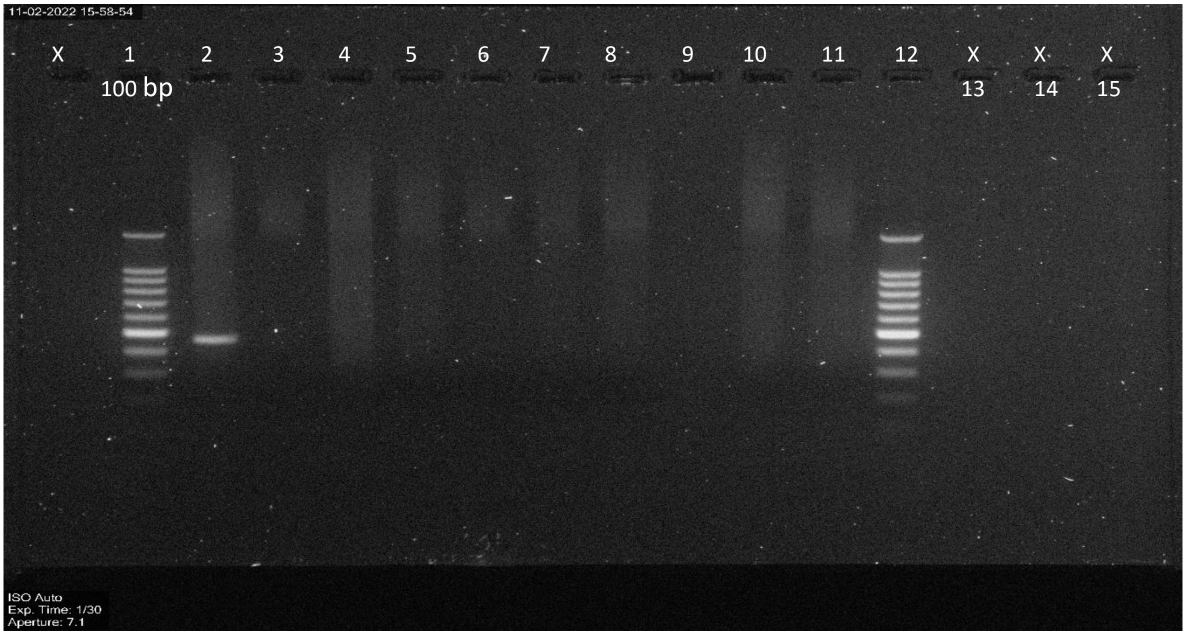

S1.3 Gel electrophoresis of monoplex PCR for *Giardia intestinalis*. Lane 1 and 12: 100 bp DNA marker. Lane 2 positive control showing a 463 bp amplicon. Lane 3: negative control. Lanes 4 – 11 negative samples. Lanes 13-15, and/or with a cross, are empty. Capture method: Gel Doc system LED FastGene FAS-DIGI PRO (NIPPON Genetics Europe). Lanes 1-12 were included in Appendix 1, Fig S1.3.

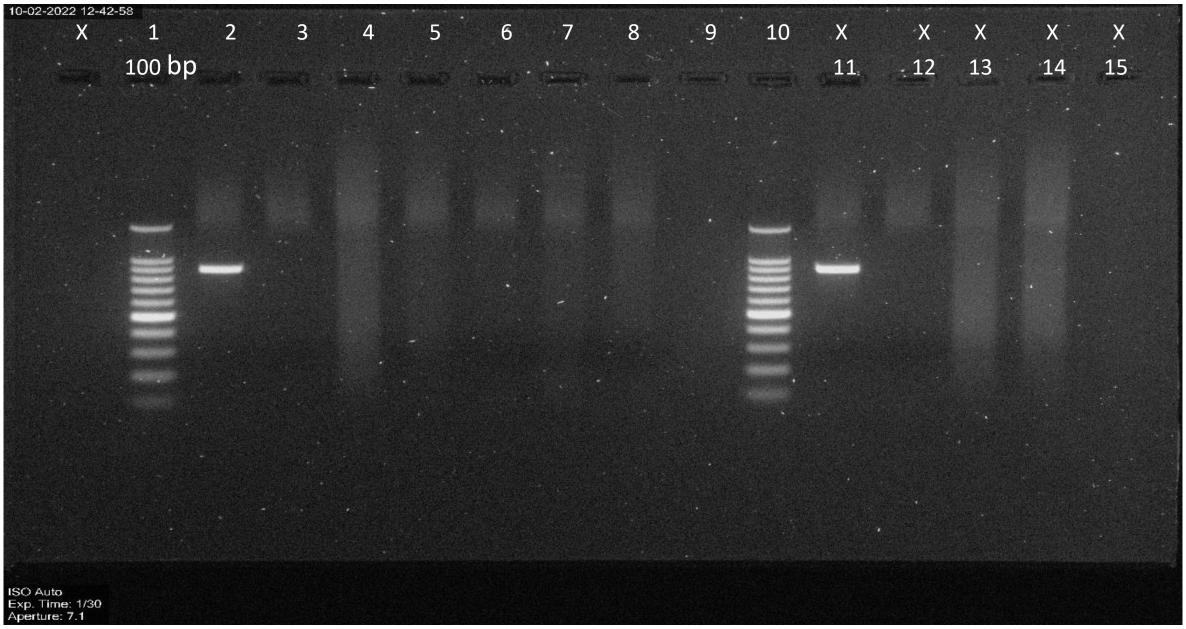

S1.4 Gel electrophoresis of monoplex PCR for *Dientamoeba fragilis*. Lane 1 and 10: 100 bp DNA marker. Lane 2 and 11 are positive controls (synthetic DNA) showing a 850 bp amplicon. Lane 3: negative control. Lanes 4 – 8 and 12-14 negative samples. Lanes 9 and 15 are empty. Capture method: Gel Doc system LED FastGene FAS-DIGI PRO (NIPPON Genetics Europe). Lanes 1-10 were included in Appendix 1, Fig S1.4.

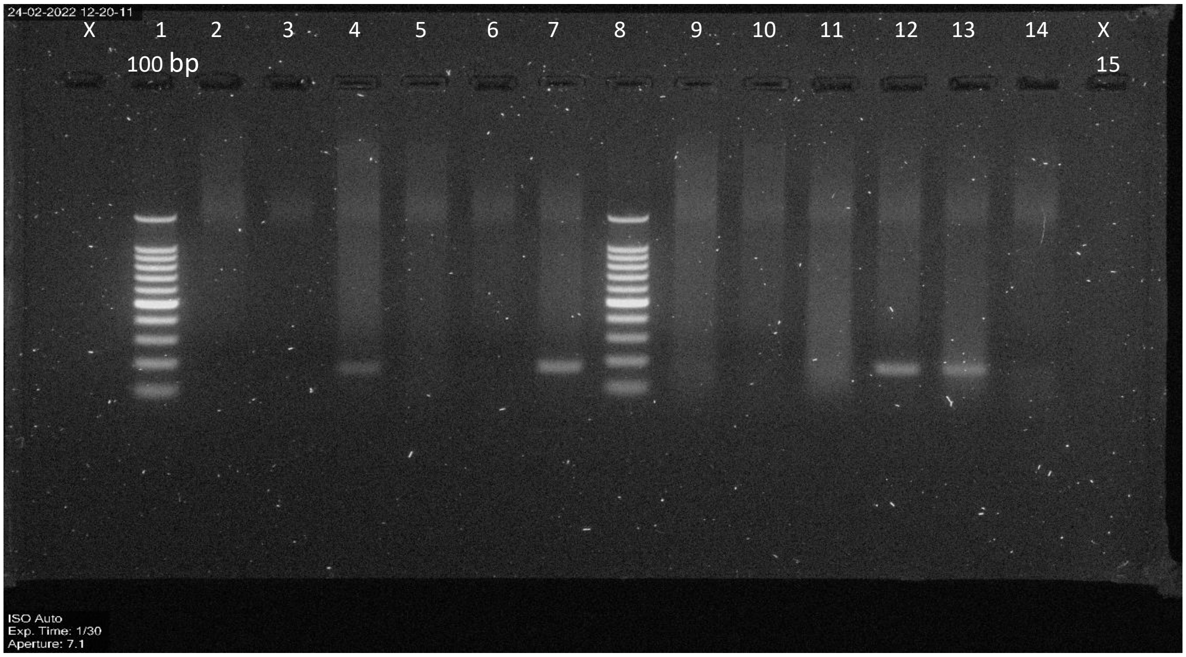

S1.5 Gel electrophoresis of monoplex PCR for *Entamoeba dispar*. Lane 1 and 8: 100 bp DNA marker. Samples: Lanes 3-7 and 9-14. Lane 2 negative control. Positive samples: lanes 4, 7, 12 and 13 show a 174 bp amplicon. Negative samples: 3, 5, 6, 9-11, 14. Lane 15: empty. Capture method: Gel Doc system LED FastGene FAS-DIGI PRO (NIPPON Genetics Europe). Lanes 1-14 were included in Appendix 1, Fig S1.5.
