# Supplementary material for: Molecular diagnosis of intestinal protozoa in young adults and their pets in Colombia, South America
Source: PLoS One. 2023 May 23;18(5):e0283824. doi: 10.1371/journal.pone.0283824 (PMC10204978; doi:10.1371/journal.pone.0283824)
Supplement: Attachment — Submitted filename: Response to Reviewers.pdf [file pone.0283824.s004.pdf]

October 20th, 2022

To the Editor(s) of
PLOS One

Dear sir (s),

Thank you for your helpful comments regarding our manuscript: "Molecular diagnosis of intestinal protozoa in young adults and their pets in Colombia, South America".

I would like to request a change in the author´s order as follows:

Caterine Potes-Morales - first author

Maria del Pilar Crespo-Ortiz - second author

I apologize for the incovenience.

Please find below a point-by-point response to editors and reviewers.

Editor and Reviewer comments:

Journal Requirements:

When submitting your revision, we need you to address these additional requirements.

1.Please ensure that your manuscript meets PLOS ONE's style requirements, including those for file naming. The PLOS ONE style templates can be found at https://journals.plos.org/plosone/s/file?id=wjVg/PLOSOne_formatting_sample_main_body.pdf and https://journals.plos.org/plosone/s/file?id=ba62/PLOSOne_formatting_sample_title_authors_affiliations.pdf

Authors: We have revised the style requirements and renamed the supporting information files and relocated this section after the references. Other requirements have been also fixed (tabs, figures, tables, and general formatting).

2. Thank you for stating the following in the Acknowledgments Section of your manuscript:

"We also acknowledge Meleny Ramirez and Claudia Auseche (Department of Microbiology, University del Valle) for assistance with the microscopy work."

We note that you have provided funding information that is not currently declared in your Funding Statement. However, funding information should not appear in the Acknowledgments section or other areas of your manuscript. We will only publish funding information present in the Funding Statement section of the online submission form.

Please remove any funding-related text from the manuscript and let us know how you would like to update your Funding Statement. Currently, your Funding Statement reads as follows:

"This research was financially supported by the Internal Grant Scheme 2020 (CI1920) awarded to CPM and MPC by the Universidad del Valle, Cali, Colombia. The funder had no role in study design, data collection and analysis, decision to publish, or preparation of the manuscript."

Please include your amended statements within your cover letter; we will change the online submission form on your behalf.

Authors: We have modified the acknowledgements section to clarify the information. We have declared in the financial disclosure our funding as it is stated in the above paragraph (as in submission), we have no changes to report or further funding sources to declare.

3. We note that Figure 1 in your submission contain map images which may be copyrighted. All PLOS content is published under the Creative Commons Attribution License (CC BY 4.0), which means that the manuscript, images, and Supporting Information files will be freely available online, and any third party is permitted to access, download, copy, distribute, and use these materials in any way, even commercially, with proper attribution. For these reasons, we cannot publish previously copyrighted maps or satellite images created using proprietary data, such as Google software (Google Maps, Street View, and Earth). For more information, see our copyright

Authors: Figure 1 has been replaced by a figure made by our collaborators and free of copyright restrictions.

Reviewers' comments:

Reviewer #1: The Graph (s) presentation is missing, some selective addition of tables. There may be inclusion of broad area study including various municipalities and districts and more study focus on old age subjects or host owners and immuno-compromised persons.

Authors: We have verified that figures and tables are cited properly through the manuscript. We agree that studies in other population groups should be included for surveillance particularly, those in host owners. We focused our study in young adult population where the data on intestinal parasites is still limited. The aim was to point out the need to include this group for surveillance in other regions and to address control interventions.

Reviewer #2: Abstract
Line 46: Suggest removing 'highly contaminated environment' and replacing with a phrase that indicates interaction and exposure to an infected animal and/or infected animal's environment.
Please include genus and species if possible.
Authors: this has been fixed.

Introduction
Line 55-58: References needed
Authors: References were added.

Line 58: Migration of wild animals? Please specify.
Authors: this has been fixed as "human migration"

Line 64: Please describe what Disability Adjusted Life Years is and how it is relevant.
Authors: This has been fixed as suggested.

Line 74: Describe why the range is so large for *Dientamoeba* infections
Authors: this has been fixed.

Line 79: High variability due to what factors?
Authors: Factors have been added.

Materials and Methods:

Line 111: Selection criteria for students? Describe clinical variables. Was any data excluded for a particular student? If so, please justify.
Consider including references for microscopy methods that have been described as appropriate.

Authors: this has been fixed as suggested.

Table 1. Should be thermocycling, not termocycling.

Authors: fixed.

Statistical Analysis: Did you evaluate any interactions between variables?

Authors: Yes, a binomial logistic regression was used to evaluate the determinant factors for parasite infection. Demographic and behavioral variables were included as independent variables and the parasitological status was used as a dependent variable. The model only showed that parasite infection was associated with participants enrolled in the health care assistance program (SISBEN). This association was found in the bivariate analysis, so the regression results were not included.

Results

Line 87: Include n number consistently

Authors: This has been fixed in the whole manuscript.

Table 2. Include total n number for positive and negative. Describe social assistance program since this is the only variable where significance was observed. Remove the footnote about having more than one pet or clarifying for the other similar variables as well. Consistency is key.
Again, please be consistent with n numbers, etc. throughout the entire manuscript.

Authors: n numbers have been added when indicated. A description of the social program has been added and the table footnote has been removed.

Table 3. Why does the n number differ across the floats and staining technique?

Authors: In several cases, insufficient sample volume was provided to perform flotation or trichome techniques.

Please describe how you differentiated between the protozoa based on morphology or include reference.
Please include zeros or NA in the table if you did not detect or evaluate.

Authors: fixed.

Line 250: How did you determine overall prevalence? Clarify please. Include n number for reference.

Authors: fixed.

Did you have more false positives with microscopy or PCR?

Authors: We had 10 cases of *Entamoeba* complex by microscopy that in the routine lab could be judged presumptively as *E. histolytica*, however none of them were positive by PCR, so we could consider those as false positives. Nevertheless, to better determine the rate of false positives for microscopy and PCR, a different technique should be included. For instance: two cases of *Giardia* were not detected by PCR, but the morphology by microscopy was typical of this protozoon, so we can´t consider this as a false positive. *Cryptosporidium* amplicons obtained by PCR were verified by sequencing, however *Blastocystis* amplicons were not subjected to sequencing due to limited funding.

Do you think the DNA extraction method used reduced detection of *Endolimax nana* by PCR?

Authors: In our study we did not perform PCR for *E. nana*.

Line 263-264: Split into two sentences.

Authors/fixed

Table 5. Provide more information to help reader interpret this data. It is not straightforward.

Authors: The table has been modified and more information has been added in the text.

Supplementary Gel Image 2. Is there another band 105 -110bp?

Authors: Some very weak bands are seen below 100 bp, these likely are remaining primers from the master mix.

Reviewer #3: It was an extensive search. It is remarkable that parasites, which constitute an important public health problem, have been identified. I also consider the coexistence of several parasites to be a valuable finding. It is important for me to answer the parts I have explained below. I also think that some corrections will make the article more fluent and understandable.
The first thing that caught my attention is why T. gondii infections, one of the serious zoonotic infections, are ignored. Should have been included in this study in some way. However, Echinococcus gronulosus could also be investigated. should not be restricted to protozoal infections only.
Its importance in humans and animals should be considered in more detail. As a result, a unilateral infection does not occur.
The charts are nice, but a separate coloured chart with co-infections would be more effective. It will be more understandable if the work in a more graphic style is strengthened.

Authors: The aim of our study was the diagnosis of the most frequent parasites in humans and to know the prevalence in a young adult population. Samples were examined for protozoa and helminths by microscopy, but helminths were not found. Molecular analysis focused on protozoans as they were the most frequently found and due to limited funding.

We have added a comment about our *Toxoplasma* findings in the discussion section. We agree with the reviewer about the impact of *T. gondii* infections, therefore the relevant findings in our study represent a starting point for further research. We expect to apply for funding to make a more extensive molecular analysis in human and animal samples including those for *Toxoplasma* and helminths.

We have modified and split the Figure 2 (parasite coinfections) to make it clearer.

Following the reviewer´s suggestions the manuscript has been fully revised to make it more fluent and understandable.

Sincerely,

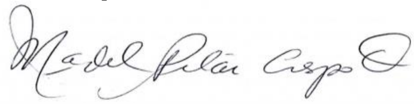

Maria del Pilar Crespo-Ortiz
MSc, PhD
Professor
Department of Microbiology
University del Valle
Cali, Colombia.
