# Supplementary material for: Molecular diagnosis of intestinal protozoa in young adults and their pets in Colombia, South America
Source: PLoS One. 2023 May 23;18(5):e0283824. doi: 10.1371/journal.pone.0283824 (PMC10204978; doi:10.1371/journal.pone.0283824)
Supplement: Attachment — Submitted filename: Response to editor and rewiever.docx [file pone.0283824.s005.docx]

February 21^st^, 2023

To the Editor(s) of

PLOS One

Dear Editor,

Thank you for your helpful comments regarding our manuscript: “Molecular diagnosis of intestinal protozoa in young adults and their pets in Colombia, South America”.

**Please find below a point-by-point response to editors and reviewer.**

**To Editor comments:**

Please respond to reviewer number one

**Authors: We have carefully read the reviewer # 1´s comments. The observations are too general, but we have added some information expecting to fulfill all the requirements and suggestions. We have observed that after the first round of evaluation all the reviewers agreed with questions #1 to #5, however, in the last assessment the reviewer #1 changed the answers in questions #2, #3 and #4.**

**Regarding this we have shown in detail all methods and the supporting information, including the raw gel images. The data analysis was conducted using SPSS v 27 and results were also verified in Epi info v 7.2.5. In the manuscript (Statistical analysis section) we have also included the multiple logistic regression analysis which further support our results.**

**To Reviewer comments:**

Reviewer #1: A thorough study to be conducted. Missing graph and proper discussion with meager latest references.

**Authors: We have added three figures highlighting our results (Fig 1) and showing in more detail our data and the analysis performed (Fig 2 and 3). To improve the discussion section, we have included Table 6 comparing our data with published studies in similar young adult populations worldwide.**

**The references were revised and updated as far as not many published studies have been conducted in student young adults.**

**As we stated before we agree that studies in other population groups should be included for surveillance particularly for *Cryptosporidium*, those studies may correspond to future research. We have provided the starting point for this research and reinforce the need for molecular testing. The presented work has focused on young adult population because the data on intestinal parasites is scarce, and the effects of parasite infection are unknown. In our latest literature review we could not find a study with a similar approach in the young adult population and exploring the involvement of animal exposure.**

Sincerely,

Maria del Pilar Crespo-Ortiz

MSc, PhD

Professor

Department of Microbiology

University del Valle

Cali, Colombia.
