# Supplementary material for: Molecular diagnosis of intestinal protozoa in young adults and their pets in Colombia, South America
Source: PLoS One. 2023 May 23;18(5):e0283824. doi: 10.1371/journal.pone.0283824 (PMC10204978; doi:10.1371/journal.pone.0283824)
Supplement: Attachment — Submitted filename: Response to editor 090323.docx [file pone.0283824.s006.docx]

March 9^th^, 2023

To the Editor(s) of

PLOS One

Dear Editor,

Thank you for your helpful comments regarding our manuscript: “Molecular diagnosis of intestinal protozoa in young adults and their pets in Colombia, South America”.

**Please find below a point-by-point response to the Academic Editor**

**To Editor comments:**

ACADEMIC EDITOR: Please write a proper discussion and include more recent references. Delete this map and replace it with a more accurate one with a scale. Avoid using the Google map.

**Authors:**

1. **All the manuscript and particularly the discussion has been revised. In the discussion section the following additions/revisions were performed (Line number as in file: Revised manuscript with track changes).**

- **Contrasting information of general findings with other studies. Lines 344-345 , 351-353, 355-367.**
- **Explaining and contrasting data from Table 6. Lines 388-396**
- **Adding and refining information on *Cryptosporidium*. Lines 412-431**
- **Adding and contrasting information on *Cryptosporidium* outbreaks and sequels. Lines 436-443.**
- **Discussion on recent data and adding a paragraph (*Entamoeba* complex). Lines 465-474.**
- **Refining a paragraph (Polyparasitism). Lines 475-482.**
- **Adding new information (animal contact). Lines 488-390**
- **Refining conclusion section. Lines 542- 556.**

2. **Recent references: References were updated as possible .**

**In the former revision we changed around 12 references, this time we have updated 17 references more. In total the manuscript has 89 references and now 42 are from 2020 onwards. As we stated before not many studies have been performed in young adults so we have to keep some references from studies before 2020.**

**Updated references in this revision have been highlighted in the file: Revised manuscript with track changes.**

3. **We don´t have a map in the manuscript. We removed that map a while ago. Any reference related with the map was also removed.**

**Journal requirements:**

**References**

**Authors:** List of references has been carefully revised.

**Figures:**

**Authors:** All figures were uploaded into PACE before been submitted to PLOS One.

Sincerely,

Maria del Pilar Crespo-Ortiz

MSc, PhD

Professor

Department of Microbiology

University del Valle

Cali, Colombia.
